# Gastric stricture following corrosive agent ingestion: A case report

**DOI:** 10.1016/j.ijscr.2020.09.067

**Published:** 2020-09-21

**Authors:** Gunadi, Mukhamad Arif Munandar, Aditya Rifqi Fauzi, Susan Simanjaya, Wahyu Damayanti

**Affiliations:** a Pediatric Surgery Division, Department of Surgery, Faculty of Medicine, Public Health and Nursing, Universitas Gadjah Mada/Dr. Sardjito Hospital, Yogyakarta, 55281, Indonesia; b Department of Child Health, Faculty of Medicine, Public Health and Nursing, Universitas Gadjah Mada/Dr. Sardjito Hospital, Yogyakarta, 55281, Indonesia

**Keywords:** Corrosive agent, Endoscopic examination, Gastric outlet obstruction, Gastrojejunostomy and Braun anastomosis, Gastric stricture, Pediatric case

## Abstract

**Introduction:**

Gastric stricture due to corrosive ingestion is a rare cause of obstruction in the upper gastrointestinal tract in children. However, only a few reports highlight the management of the stricture of the gastric antrum and pylorus, i.e. gastric outlet obstruction (GOO) due to corrosive ingestion, particularly in children.

**Presentation of case:**

We report a 1-year-old male who presented with chief complaints of upper abdominal pain and profuse vomiting after accidentally ingesting sulfuric acid one month prior. On physical examination, minimal epigastric distension was found. Endoscopic examination showed oesophagitis, erosive gastritis, multiple gastric ulcers and suspicion of pyloric stricture. We decided to perform an exploratory laparotomy and found severe strictures from the major curvature to the gastric pylorus. Subsequently, we conducted gastrojejunostomy and Braun anastomosis. The patient was discharged on the eighth postoperative day uneventfully.

**Discussion:**

Endoscopy is a reliable technique for assessing upper digestive tract mucous membranes after caustic agent ingestion because it helps in making a definitive diagnosis, especially to define the anatomic location and injury severity. The timing and type of surgery for GOO is controversial. We performed Braun anastomosis in addition to gastrojejunostomy because of several advantages over other surgical methods.

**Conclusion:**

We recommend choosing an appropriate supporting examination to precisely diagnose gastric stricture due to corrosive ingestion. Gastojejunostomy and Braun anastomosis show a good outcome for gastric stricture due to corrosive ingestion, particularly in children.

## Highlights

• Different caustic agents will have a different impact on tissue damage and specific organs.
• Appropriate supporting examination is necessary to precisely diagnose gastric stricture due to corrosive ingestion.
• Gastrojejunostomy and Braun anastomosis is a good option for gastric outlet obstruction due to corrosive ingestion.

## 1 Introduction

Gastric stricture due to corrosive agent ingestion is a rare cause of obstruction in the upper gastrointestinal tract in children [1]. These injuries are still increasing in developing countries, relating to social, economic, and educational variables, mainly due to the lack of prevention [1]. The pediatric population represents 80% of cases of injury due to ingestion, mainly due to accidental ingestion [1]. However, only a few reports highlight the management of the stricture of the gastric antrum and pylorus, i.e. gastric outlet obstruction (GOO) due to corrosive ingestion, particularly in children [[2], [3], [4]]. This research work has been reported in line with the SCARE checklist [5].

## 2 Presentation of case

**Fig. 1:**
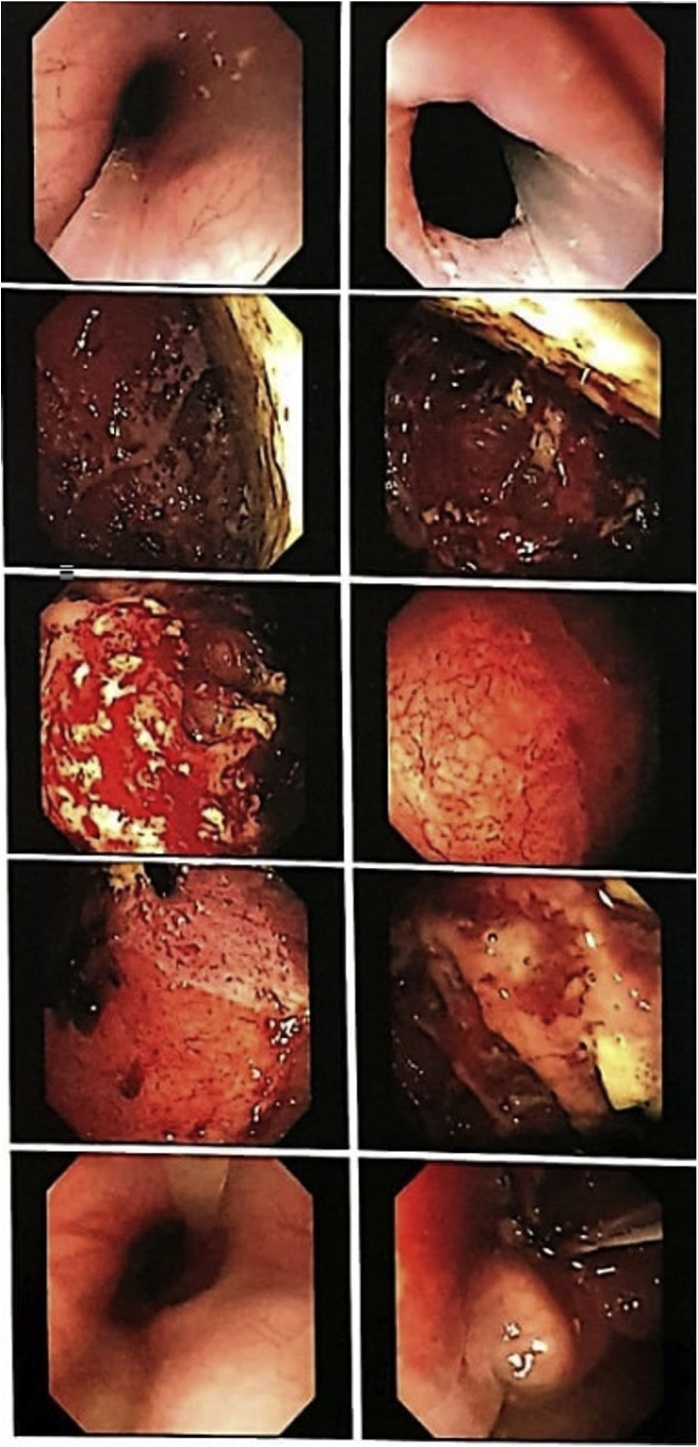
Endoscopy showed esophagitis, multiple gastric ulcers, erosive gastritis, and suspected pylorus stricture.

**Fig. 2:**
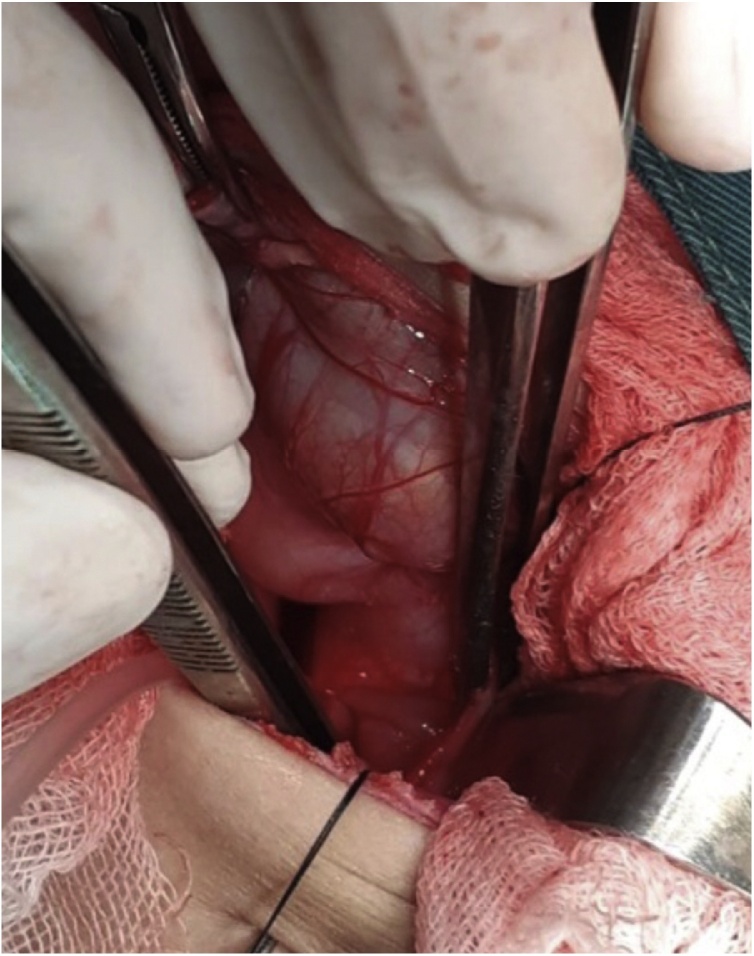
Intraoperative findings revealed severe strictures from the major curvature to the gastric pylorus.

**Fig. 3:**
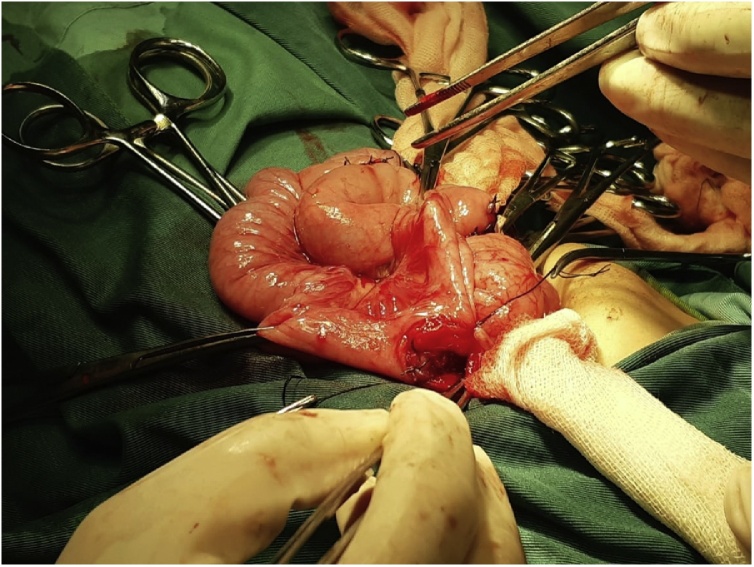
Gastrojejunostomy and Braun anastomosis were performed for the patient.

One-year-old male presented with chief complaints of upper abdominal pain and profuse vomiting after accidentally ingesting sulfuric acid one month prior. On physical examination, minimal epigastric distension was found. During the one month before admission to our hospital, the patient was treated conservatively at another hospital, including correcting nutritional deficiencies; however, it was not successful. We performed endoscopic examination and found esophagitis, erosive gastritis, multiple gastric ulcers and pyloric stricture (Fig. 1). We decided to perform an exploratory laparotomy and found severe strictures from the major curvature to the gastric pylorus (Fig. 2). Subsequently, we conducted gastrojejunostomy and Braun anastomosis (Fig. 3). The patient was uneventfully discharged on the eighth day postoperatively. There were no complications or reports of recurrence of symptoms until the last follow-up (approximately 1 year after surgery) when calling the parents during the preparation of this case report.

## 3 Discussion

Here, we report a case of gastric stricture due to accidental acid ingestion in a child. This finding is compatible with a previous report that indicated that most aetiology of ingestion injury is accidental ingestion [1]. In contrast, most ingestion injuries in adults are due to suicidal intention [1]. Our case accidentally ingested sulfuric acid. As shown by a previous report, acid injuries are more common in developing countries [1]. Acid and alkaline substances have different effects on tissue damage. Acid causes coagulation necrosis and eschar formation, whereas alkaline conditions result in liquefactive necrosis and saponification [1]. This child developed a severe stricture of the gastric antrum and pylorus, i.e. GOO. A previous study showed that acidic caustic agents cause gastric burns, particularly the antrum and pyloric parts of the stomach, while alkaline ingestion causes oesophageal burn [6]. The specific viscosity and gravity of corrosive acids are lower than those of liquid alkalis. Antral spasms cause pooling of corrosive material, resulting in more damage to the antrum. The degree of mucosal damage depends on the nature of the agent, its amount and concentration, as well as the amount of food in the stomach during ingestion [2].

Interestingly, most patients who suffer from caustic agent ingestion are children younger than 5 years [6], similar to our patient.

Endoscopy is a reliable technique for assessing upper digestive tract mucous membranes after caustic agent ingestion because it helps in making a definitive diagnosis, especially to define the anatomic location and injury severity [6]. Moreover, endoscopy is indicated for infants and children with suspected gastroduodenal obstruction if radiologic studies are uncertain or if endoscopic therapy is suggested [7]. The timing for endoscopy is still controversial. One report suggested that endoscopy is performed within 24 h after ingestion because after one day the endoscope may perforate the digestive tract [6], while another proposed that endoscopy is safe to be performed between 1–2 weeks after ingestion [1]. Our patient underwent endoscopy one month after the incident without any complications. Furthermore, imaging with computerized tomography (CT) scans has not been recommended to replace endoscopy because of its low sensitivity [8].

The timing and type of surgery for GOO is still debatable [1]. One report advised early surgery to decrease morbidity and mortality, while another suggested performing surgery more than 3 months after ingestion due to gastric wall edema, poor nutritional status and adhesion [1]. There are several techniques to treat GOO, including gastrojejunostomy, which is suggested for cases with poor general condition and large perigastric adhesion [1]. Other studies proposed Bilroth 1 as the preferred surgical approach for GOO due to acid ingestion because they hypothesize that the remaining nonviable gastric tissue will develop metaplasia, cancer or late marginal ulcers if resection is not done [2,9]. Endoscopic balloon dilation has also been suggested for the treatment of GOO, but this procedure is proposed only for temporary substitution of surgical resection since the fibrosis of the gastric wall often reduces the long-term functional result [1]. Although pyloroplasty is recommended for moderate stricture, progressive fibrosis often results in recurrent stricture [1].

Our case underwent gastrojejunostomy and Braun anastomosis because of severe stricture of the gastric antrum and pylorus approximately one month after the incident of accidental caustic agent ingestion and showed good outcome. We performed Braun anastomosis in addition to gastrojejunostomy because of several advantages as follows: it decreases the incidence of delayed gastric emptying, prevents the possibility of twisting, angulation, kinking and edema of gastrojejunostomy, enables the route of the food, reduces the possibility of gastric mucosa exposure from bile and pancreatic juice reflux, and decreases the incidence of loop obstruction [10].

In addition, we performed the surgery in patients with malnourished nutritional status. Therefore, there is a possibility of leakage of anastomosis since one of the factors affecting the anastomosis healing process is nutritional status [11]. Fortunately, our patient was uneventfully discharged on the eighth day postoperatively, and no complications or reports of recurrence of symptoms were reported until the last follow-up (approximately 1 year after surgery). This might be associated with the evidence that there are many factors affecting anastomotic leakage, not only patients’ nutritional status [12].

## 4 Conclusions

We recommend choosing an appropriate supporting examination to precisely diagnose gastric stricture due to corrosive ingestion. Gastrojejunostomy and Braun anastomosis show a good outcome for gastric stricture, i.e. GOO due to corrosive ingestion, particularly in children.

## Declaration of Competing Interest

The authors report no declarations of interest.

## Funding

The authors declare that this study had no funding source.

## Ethical approval

The informed consent form was declared that patient data or samples will be used for educational or research purposes. Our institutional review board also do not provide an ethical approval in the form of case report.

## Consent

Written informed consent was obtained from the parent of the patient for publication of this case report and accompanying images. A copy of the written consent is available for review by the Editor-in-Chief of this journal on request.

## Author’s contribution

Gunadi conceived the study. Gunadi, Mukhamad Munandar, Aditya Rifqi Fauzi, Susan Simanjaya drafted the manuscript. Wahyu Damayanti critically revised the manuscript for important intellectual content. Gunadi, Mukhamad Munandar, Aditya Rifqi Fauzi, Susan Simanjaya and Wahyu Damayanti facilitated all project-related tasks.

## Registration of research studies

The manuscript is a case report, not considered a formal research involving participants.

## Guarantor

Gunadi.

## Provenance and peer review

Not commissioned, externally peer-reviewed.
